# How Females Keep Male Hoverfly Visual Neurons from Distraction

**DOI:** 10.1371/journal.pbio.0040081

**Published:** 2006-02-07

**Authors:** Jami Milton Dantzker

Legendary athletes like running back Gale Sayers and center fielder Willie Mays earned fame and fortune by pursuing a target traveling at high speeds against a moving, chaotic background. But most animals rely on this evolutionarily honed skill for more fundamental needs. Peregrine falcons snag prey while flying at speeds approaching 100 miles per hour. Males from several fly species chase females during dazzling courtship dances with impressive aerial maneuvers. How the brain accomplishes visual pursuit has been a longstanding question, which becomes even more fascinating when one considers how the tiny nervous system of the male fly executes the visual precision and flight control necessary during courtship chase behavior.

The visual system of the male fly lends an attractive model for investigating this question, partly because females don't chase males during courtship, allowing scientists to link the male-specific neurons to behaviorally relevant functions. In a new study, Karin Nordström, Paul Barnett, and David O'Carroll present surprising insight into the neural basis of visual pursuit in male hoverflies *(Eristalis tenax)*, and identify a novel class of neurons associated with the task.

While hoverflies responded to pixel-sized dots moving against a cluttered, moving background, the authors measured neural activity in the hoverfly visual system (called the lobula complex). Even though the distracting background stimulus sometimes moved at the same velocity as the target, one class of neurons showed highly specialized tuning properties for the targets. The authors refer to these neurons as “small target motion detectors” (STMD). For a sense of the neurons' performance, imagine driving a golf ball high up into a partly cloudy sky and trying to keep your eye on it while it soars through the air. The hoverfly neurons might be able to track the golf ball even if the sky was moving at the same speed as the ball.

To get this intriguing data, Nordström et al. secured 74 wild-caught male hoverflies in front of a monitor and presented the flies with a range of target and background stimuli that varied in contrast, movement, direction, and speed. The authors recorded intracellular electrical activity from several hundred individual neurons. Of these, 206 neurons shared the striking sensitivity to small moving targets, and the authors classified them as STMD neurons.

To further characterize the STMD neurons' response properties, the authors varied the speed of the background motion. They expected the moving background to suppress target response based on the “feedback hypothesis,” which predicts that target neuron response would be dampened by the surrounding inhibitory circuitry. Unexpectedly, most STMD neurons maintained their sensitivity to the targets despite the distracting background motion. The authors also empirically confirmed the absence of inhibition in one class of STMD neurons.

To further understand the basis of the tuning properties of these STMD neurons, Nordström et al. explored the contribution of relative contrast between the target and background on the neurons' response profile. This class of STMD neurons, they found, displayed a striking sensitivity to contrast. The neurons even responded when the luminance of the background and target was similar, though the response was attenuated. This result suggests that even a slight difference in contrast is enough to evoke a response, and that when a female hoverfly moves against foliage with similar luminance properties as her body, the male's STMD neurons might be able to track her.

From the perspective of object recognition, the robust rejection of background clutter in favor of the target suggests that STMD neurons may be true feature detectors faithfully tuned to their preferred stimulus. Although predicted to exist, neurons that display such remarkable selectivity have not been particularly forthcoming. A compelling future direction of this work will be to uncover how the neural circuits of the insect compound eye and visual system mediate such exquisite feature detection. Eventually, the authors hope to link their physiological studies of visual neurons to the complex social behaviors of hoverflies.

## 

**Figure:**
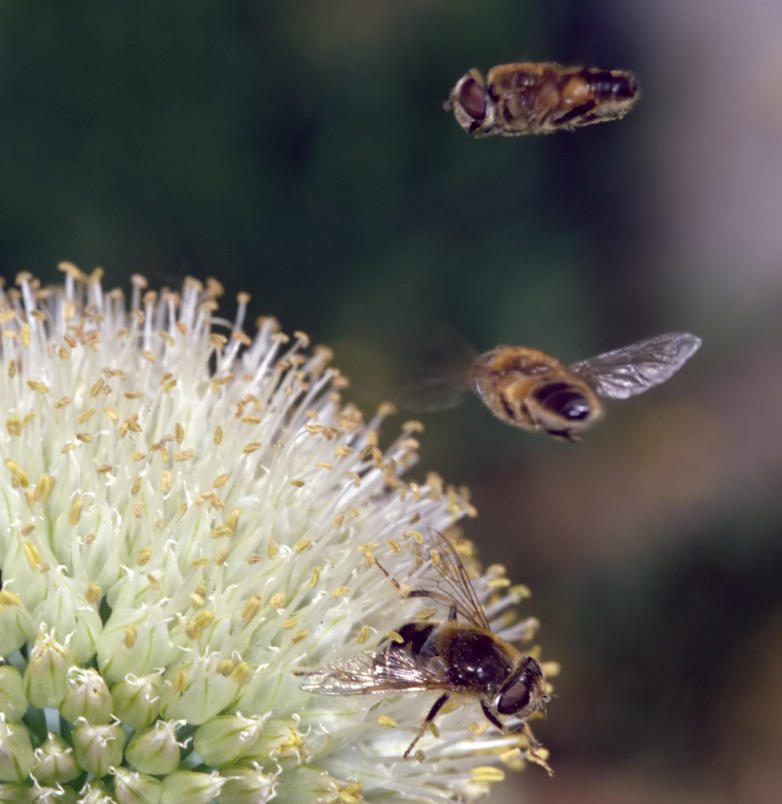
A novel class of visual neurons helps hoverflies stay focused on their target as they travel in complex visual environments. (Photo: Roman Prokhorov)

